# Factors influencing use of modern contraception among married women in Ho West district, Ghana: descriptive cross-sectional study

**DOI:** 10.11604/pamj.2019.33.15.17500

**Published:** 2019-05-10

**Authors:** Precious Afriyie, Elvis Enowbeyang Tarkang

**Affiliations:** 1 Department of Population and Behavioural Science, School of Public Health, University of Health and Allied Sciences, PMB 31, Ho, Ghana; 2 HIV/AIDS Prevention Research Network Cameroon (HIVPREC) Kumba, Kumba, Cameroon

**Keywords:** Modern contraception use, Ho West district, Ghana, married women

## Abstract

**Introduction:**

the use of modern contraception helps couples and individuals realize their basic right to decide freely and responsibly if, when and how many children to have. The current study assessed the factors influencing the use of modern contraception among married women in Ho West District, Ghana.

**Methods:**

the study adopted a descriptive cross-sectional design, using a standardized validated pretested interviewer-administered questionnaire adapted from previous studies to collect data from a systematic sample of 225 married women and analyzing them using Stata version 14 software program at the level 0.05.

**Results:**

the majority, 202 (89.8%) had used modern contraception before, and the proportion currently using some form of family planning (FP) was 130 (64.4%), majority (46.2%) of whom were currently using injectable. Majority (66.1%) used modern contraception in order to ensure proper care of children. Most (64.2%) of the women who were not using modern contraception were not doing so because of their partner's disapproval. Private employees were 0.20 times less likely to use modern contraception (AOR=0.20 (95% CI: 0.04-0.91); p=0.038) compared to housewives, while women who did not have problems with decision-making were 4 times more likely to use modern contraception (AOR=4.40 (95% CI: 1.25-14.44); p=0.021) compared to their counterparts who had problems with decision-making at home.

**Conclusion:**

the use of modern contraception is low. Health promotion interventions to increase modern contraception use among married women in Ho West District of Ghana should focus on the privately employed and those with problems in decision-making at home.

## Introduction

Modern contraception is an integral component of reproductive health and has positive effects on the health of women. Conception can take place at any time during the fertile period when fertilization occurs naturally free of any impediments (unprotected coitus) and spermatozoon is mature and capacitated. But sometimes there is need for protection against unplanned pregnancy, which may lead to unpleasant consequences [1]. A high fertility rate has been associated with poor child and maternal health as well as increased risk of maternal mortality. Despite technological advancements in modern contraceptive methods, still unintended pregnancy is a worldwide problem that affects women, their families and the society as a whole [2]. The sustainable development goals (SDGs) aim at promoting universal access to sexual and reproductive health (SRH) services [3, 4], with promotion of modern contraception being one of the main targets [3, 5]. Modern contraception improves health through adequate spacing of birth, avoiding pregnancy at high-risk maternal age and high parity [6]. Modern contraception can be broken down into barrier methods (condoms or cervical cap), hormonal methods (the pill), intrauterine devices (IUD) and sterilization. The method chosen depends on the woman's general health, lifestyle and relationships [1]. Despite these varieties, the rate of population growth and unplanned pregnancies is still high in sub-Saharan Africa (SSA) and globally [7]. A rapid population growth resulting from non-use of modern contraception is a burden on the resources of many developing countries, where unregulated fertility, compromises the economic development and political stability [7]. It was projected in 2004 that Ghana will achieve a modern contraception prevalence rate of 28% by 2010 and 50% by 2020. However, records available indicate that the current modern contraception acceptance rate in Ghana is 23%, five percentage points below the expected projection for 2010 [8].

Modern contraception and reproductive health services are sparse and vary greatly across different regions in Ghana, especially in rural and low developing population. The trend in modern contraception acceptor rate in the Volta region of Ghana in which Ho West district is found, shows a decrease from 27.8% in 2014 to 26.5% in 2016 [9]. This low rate is likely to expose women to unplanned pregnancies, inadequate child spacing and increased risks associated with closed-spaced pregnancies and child birth. The total fertility rate for the Ho West district of Ghana is 3.6 with a general fertility rate of 104.6 births per 1000 women aged 15-49 years, which is higher than the Volta region's rate of 99.2. The Crude Birth Rate (CBR) is 24.2 per 1000 population [10]. These rates will lead to a rapid population growth in Ho West district without the use of modern contraception, which will place a burden on the meagre resources of the country as a whole. Understanding the factors that influence modern contraception use is key to reducing the disparities that exist in its uptake and levels of unmet needs for modern contraception in Ho West district. Despite the promotion of modern contraception worldwide, usage has been quite low. Knowledge, religious beliefs, occupation, financial considerations, educational level, fear of side effects, partner's disapproval and problems with decision-making at home are the major factors influencing use of modern contraception [1, 11]. In view of the relatively low modern contraception acceptor rate in the Volta region of Ghana [9] and limited evidence on factors that influence use, an assessment of the current modern contraception use levels and factors that influence its use among married women in the Ho West district is timely and appropriate. The current study therefore assessed the factors that influence utilization of modern contraception among married women in Ho West district, Ghana.

## Methods

**Study site description:** Ho West district is one of the 25 districts in the Volta region of Ghana. It was established by the Legislative Instrument (LI) 2083 of 2012 and was carved out of Ho municipality in January 2012 and inaugurated in June 2013. It shares boundaries with Adaklu district to the south, Afadjato south district to the north, Ho municipality and the Republic of Togo to the east and south Dayi district to the west. It has a total land area of 1,002.79 square kilometres and a population density of 94.3 based on a population of 94,600, with males constituting 48 percent and females representing 52 percent [12]. The Ho West district has 20 health facilities of various categories (health centres, maternity homes, clinics and community-based health planning and services (CHPS) compounds), majority of which offer free family planning services [12].

**Study population:** the study population comprised married women who were between the ages of 18 and 49 years and residing in the Ho West district.

**Eligibility criteria:** women who were aged 18-49 and currently married or had been married before, mentally sound and consented to participate, were included in the study.

**Study design:** the study adopted a descriptive cross-sectional design, collecting data from a systematic sample of 225 respondents.

**Sample size determination:** the minimum sample size for the study was obtained by using Cochran formula [13]:

$$n=\frac{z^{2}pq}{d^{2}}$$

where n= sample size, Z= z score, p= estimated proportion of married women using modern contraception in Ghana = 16.7% [14]. q = 1-p, d = margin of error It was based on the assumption of a margin of error of 0.05, 95% confidence level and 0.05 (5%) non-response rate. A sample size n= {(1.96)^2 x 0.167 x (1 - 0.167)} / (0.05)^2, n= 214. Adding a non-response rate of 5%, n= (214*0.05) + 214 = 224.6. Therefore, the sample size was 225.

**Sampling method:** a systematic sampling technique was used to select the participants from all the six sub-districts that make up the Ho West district for the study (38 households from each sub-district). A community volunteer in each sub-district assisted the researchers with the community entry and in sampling the participants. All the households in each sub-district were numbered and the first household was selected at random, after which the subsequent ones were selected at an interval of 5. A total of 228 households were selected, however, three were dropped because there was no married woman in there.

**Data collection procedure:** a structured pretested interviewer-administered questionnaire was used to collect data from the respondents by the researchers, in a language they understood best. The questionnaire was developed from validated questionnaires that have been used in previous studies [15, 16] and was considered valid and reliable for use in the current study by experts. It was pretested on a convenience sample of 10 married women who did not take part in the actual study, after which it was refined for better understanding by the respondents. The questionnaire contained three sections. The first section captured information on the socio-demographics of the respondents; the second section captured information on the usage of modern contraception; the third section captured the reasons for use and non-use of modern contraception. Due to the sensitivity of the issue, only female data collectors (three) were trained for two days on how to collect data and were supervised by the researchers during data collection. Interviews were conducted in private locations with each participant at a time and maintaining interviewee privacy.

**Data analysis:** data collected were entered into EpiData version 3.1 and then exported to STATA Version 14 for cleaning and analysis. Descriptive and inferential (chi-square and logistic regressions) statistics were done to determine the relationship between the dependent variable (current usage of modern contraception contraceptive) and the independent (socio-demographic) variables. P-values less than 0.05 considered statistically significant.

**Ethical issues:** ethical approval was obtained from the Ghana Health Service Ethics Review Committee with approval number: 116/05/17. Written informed consent were sought from the participants before the commencement of data collection and participation was free.

## Results

**Socio-demographic characteristics of participants:** Table 1 depicts the demographic characteristics of the respondents. Of the 225 married women surveyed, the mean age was 32.7 ±6.1. Majority 125 (55.5%) were between the ages of 30 and 39; 95 (42.2%) had their education up to the senior high school (SHS) level; 127 (56.4%) were self-employed and 197 (87.5%) were Christians. Most, 218 (96.9%) were having children at the time of the survey, of which 141 (62.7%) had 1 to 2 children. All the children of the 218 women with children were reported to be alive at the time of the survey. The mean age at first marriage was 27.4±4.6, with majority 113 (50.2%) having gotten married between the ages of 25 and 29. Most, 100 (44.4%) got married to their husbands when the husbands were between the ages of 30 and 34. Majority, 155 (68.8%) of the women at the time of the survey had been married for the past 1-5 years and majority, 147 (65.3%) of the husbands had up to junior high school / senior high school (JHS/SHS) level education.

**Table 1:** socio-demographic characteristics of the participants (N=225)

Characteristic	Frequency (percentage)
**Age (years)**	
< 25	20 (8.9)
25-29	45 (20)
30-34	79 (35.1)
35-39	46 (20.4)
>40	35 (15.6)
Mean age (±SD)	32.7(6.1)
**Educational level of Women**	
No formal education	5 (2.2)
Primary	25 (11.1)
JHS	60 (26.7)
SHS	95 (42.2)
Tertiary	40 (17.8)
**Occupation**	
House wife	27 (12)
Government employee	41 (18.2)
Private employee	30 (13.3)
Self employed	127 (56.4)
**Religion**	
Christianity	197 (87.5)
Islam	16 (7.2)
African Traditional	12 (5.3)
Marital Information	
**Age at marriage (years)**	
< 25	49 (21.8)
25-29	113 (50.2)
30-34	42 (18.7)
35-39	21 (9.3)
Mean age at marriage (±SD)	27.4(4.6)
**Husband’s age at marriage (years)**	
< 25	17 (7.6)
25-29	42 (18.7)
30-34	100 (44.4)
35-39	43 (19.1)
>40	23 (10.2)
Mean age of husband at marriage (±SD)	32.1(6.45)
**Numbers of years married (years)**	
1-5	155 (68.8)
6-10	53 (23.6)
>10	17 (7.6)
Husband’s education level	
No formal education	34 (15.1)
JHS/SHS	147 (65.3)
Tertiary	44 (19.6)
**Number of children**	
No child	7 (3.1)
1-2	141 (62.7)
3-4	56 (24.9)
>5	21 (9.3)
**Number of children alive**	
1-2	147 (67.4)
3-4	52 (23.9)
>5	19 (8.7)
**Number of desired children**	
1-2	15 (6.7)
3-4	163 (72.4)
>5	47 (20.9)

**Figure 1:**
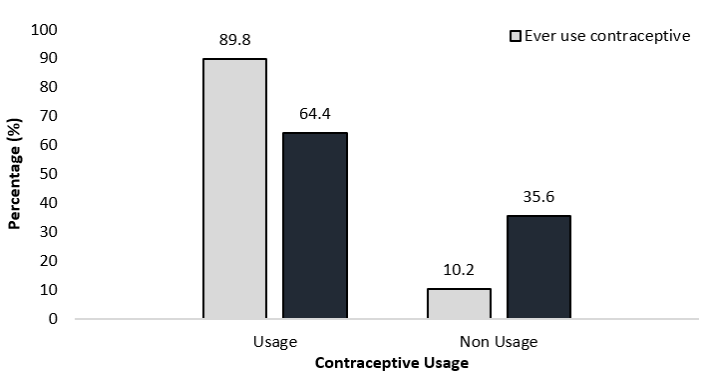
usage of modern contraception

**Figure 2:**
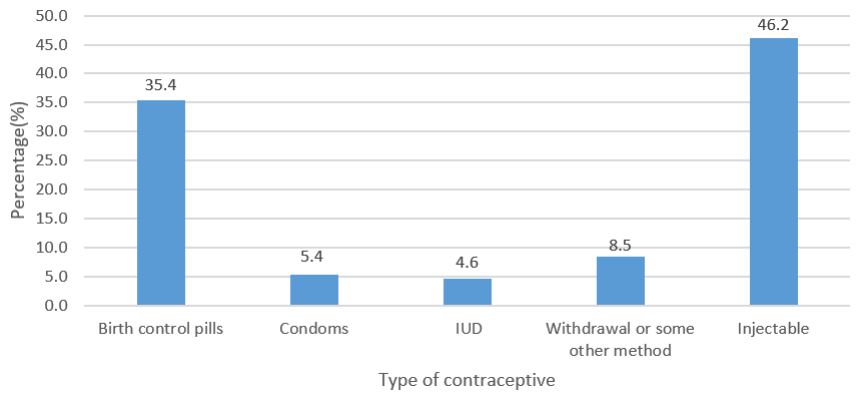
types of modern contraception used currently by participants

**Usage of modern contraception among the married women:** majority, 202 (89.8%) had used some form of modern contraception before, while 130 (64.4%) were currently using some form of modern contraceptive method (Figure 1). Of the 130 women currently using modern contraceptive methods, majority (46.2%) were using injectable followed by those using birth control pills (35.4%) (Figure 2).

**Figure 3:**
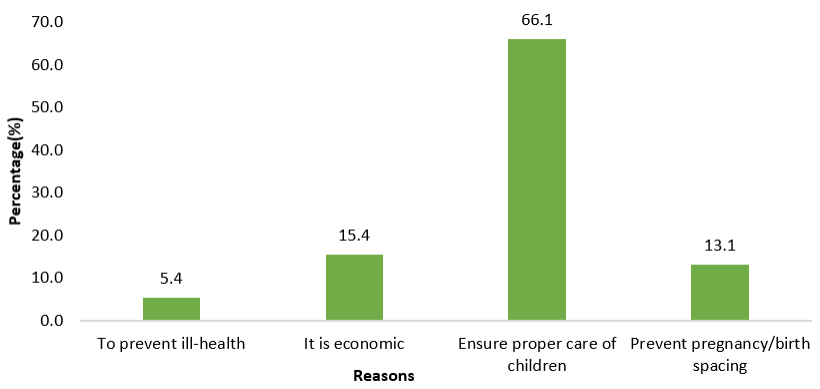
reasons for current use of modern contraception

**Figure 4:**
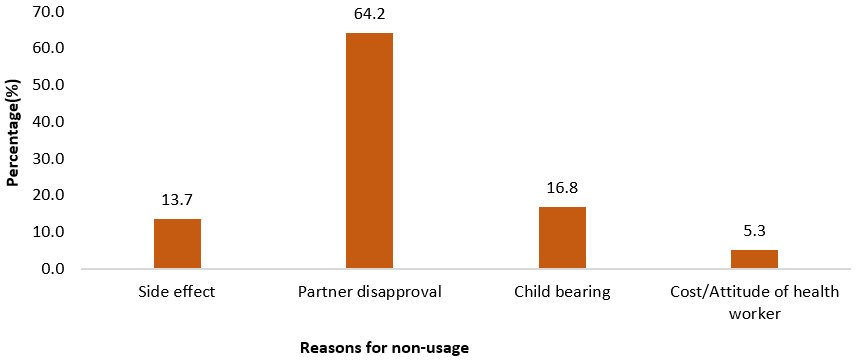
reasons for non-usage of modern contraception

**Reasons for use or non-use of modern contraception:** majority (66.1%) were using modern contraception in order to ensure proper care of children (Figure 3), while most (64.2%) were not using it because of their partner's disapproval (Figure 4).

**Associations between socio-demographic characteristics and current usage of modern contraception:** there were significant associations between age of the women, their occupation, age at marriage, problems with decision-making at home and current use of modern contraception (χ^2^=17.07, p=0.002), (χ^2^=14.79, p=0.002), (χ^2^=11.32, p=0.010) and (χ^2^=10.03, p=0.002) respectively (Table 2).

**Table 2:** association between socio-demographic characteristics and current usage of modern contraception

	Currently using any modern contraceptive method				
Characteristic	Yes n (%)	No n (%)	Total n (%)	Chi square (χ2)	P-value
**Age (years)**					
< 25	15(11.5)	3(4.2)	18(8.9)	17.07	0.002
25-29	29(22.3)	10(13.9)	39(19.3)		
30-34	33(25.4)	38(52.8)	71(35.1)		
35-39	31(23.9)	15(20.8)	46(22.8)		
>40	22(16.9)	6(8.3)	28(13.9)		
**Education**					
No formal education	2(1.5)	0(0.0)	2(1.0)	8.19	0.085
Primary	9(6.9)	9(12.5)	18(8.9)		
JHS	38(29.2)	16(22.2)	54(26.7)		
SHS	51(39.2)	38(52.8)	89(44.1)		
Tertiary	30(23.1)	9(12.5)	39(19.3)		
**Occupation**					
House wife	19(14.6)	3(4.2)	22(10.9)	14.79	0.002
Government employee	31(23.9)	9(12.5)	40(19.8)		
Private employee	12(9.2)	17(23.6)	29(14.4)		
Self employed	68(52.3)	43(59.7)	111(54.9)		
**Age at marriage (years)**					
< 25	35(26.9)	8(11.1)	43(21.3)	11.32	0.010
25-29	59(45.4)	47(65.3)	106(52.5)		
30-34	25(19.2)	15(20.8)	40(19.8)		
35-39	11(8.5)	2(2.8)	13(6.4)		
**Husband’s age at marriage (years)**					
< 25	13(10.0)	1(1.4)	14(6.9)	8.40	0.078
25-29	26(20.0)	12(16.7)	38(18.8)		
30-34	57(43.8)	35(48.6)	92(45.5)		
35-39	21(16.2)	19(26.4)	40(19.8)		
>40	13(10.0)	5(6.9)	18(8.9)		
Husband’s education					
No formal education	16(12.3)	8(11.1)	24(11.9)	5.12	0.077
JHS & SHS	81(62.3)	55(76.4)	136(67.3)		
Tertiary	33(25.4)	9(12.5)	42(20.8)		
**Number of years married (years)**					
1-5	89(68.5)	51(70.8)	140(69.3)	1.78	0.410
6-10	29(22.3)	18(25.0)	47(23.3)		
>10	12(9.2)	3(4.2)	15(7.4)		
**Number of children**					
No child	6(4.6)	0(0.0)	6(3.0)	6.02	0.111
1-2 children	80(61.5)	48(66.7)	128(63.4)		
3-4 children	31(23.9)	21(29.2)	52(25.7)		
>4 children	13(10.0)	3(4.2)	16(7.9)		
**Number of children alive**					
1-2 children	81(65.3)	50(69.4)	131(66.8)	3.88	0.144
3-4 children	30(24.2)	20(27.8)	50(25.5)		
>4 children	13(10.5)	2(2.8)	15(7.7)		
**Desired number of children**					
1-2 children	10(7.7)	3(4.2)	13(6.4)	3.86	0.145
3-4 children	98(75.4)	49(68.1)	147(72.8)		
>4 children	22(16.9)	20(27.8)	42(20.8)		
**Problems with decision-making at home**					
Yes	4(3.1)	11(15.3)	15(7.4)	10.03	0.002
No	126(96.9)	61(84.7)	187(92.6)		
Religion					
Christianity	118(90.8)	60(83.3)	178(88.1)	5.43	0.066
Islam	5(3.8)	9(12.5)	14(6.9)		
African Traditional	7(5.4)	3(4.2)	10(5.0)		

**Factors influencing the use of modern contraception:** adjusted logistic regression was conducted to determine the strength of association between factors influencing the use of modern contraception (socio-demographic factors) and its use (Table 3). In the model, occupation and decision-making significantly influenced the use of modern contraception. Women who were private employees were 0.20 times less likely to use family planning (FP) (AOR=0.20 (95% CI: 0.04-0.91); p=0.038) compared to housewives, while women without problems with decision-making at home were 4 times more likely to use modern contraception (AOR=4.40 (95% CI: 1.25-14.44); p=0.021) compared to those with problems in decision-making at home.

**Table 3:** logistic regression between current use of modern contraception and socio-demographic factors

	Currently use of modern contraceptive							
Characteristic	Yes n (%)	No n (%)	COR	95% CI	P-value	AOR	95% CI	P-value
**Occupation**								
House wife	19(14.6)	3(4.2)						
Government employee	31(23.9)	9(12.5)	0.54	0.13-2.26	0.403	0.91	0.19-4.26	0.901
Private employee	12(9.2)	17(23.6)	0.11	0.02-0.46	0.003	0.20	0.04-0.91	0.038
Self employed	68(52.3)	43(59.7)	0.25	0.07-0.89	0.033	0.411	0.10-1.65	0.207
**Age at marriage**								
< 25	35(26.9)	8(11.1)						
25-29	59(45.4)	47(65.3)	0.29	0.12-0.68	0.004	0.46	0.16-1.30	0.143
30-34	25(19.2)	15(20.8)	0.38	0.14-1.04	0.059	0.54	0.15-1.94	0.345
35-39	11(8.5)	2(2.8)	1.26	0.23-6.82	0.791	2.10	0.27-16.34	0.477
**Husband’s age at marriage (years)**								
< 25	13(10.0)	1(1.4)						
25-29	26(20.0)	12(16.7)	0.17	0.02-1.42	0.102	0.30	0.03-0.28	0.295
30-34	57(43.8)	35(48.6)	0.13	0.02-1.00	0.050	0.35	0.04-3.35	0.361
35-39	21(16.2)	19(26.4)	0.10	0.01-0.71	0.023	0.20	0.02-2.14	0.184
40+	13(10.0)	5(6.9)	0.20	0.02-1.96	0.167	0.27	0.02-3.52	0.316
**Problems in decision making**								
Yes	4(3.1)	11(15.3)						
No	126(96.9)	61(84.7)	5.68	1.74-18.57	0.004	4.40	1.25-14.44	0.021

## Discussion

Modern contraception was introduced with the aim of controlling population growth. Over the years, its use in marriage has been substantially ignored and viewed unnecessary in many parts of the world. This study specifically focused on factors influencing modern contraception use among married women resident in the Ho West district of Ghana. In the current study, current use of modern contraception among married women was 64.4%. This is higher than that of the 2014 Ghana Demographic and Health Survey (GDHS), (26.7%) [17]. This difference could be due to sensitization campaigns regarding modern contraception among married women in the Ho West district. The current finding is also higher than those of other countries, 43% in Ethiopia [16], 12.5% in Nigeria [18] and 10% in SSA in general [19]. The difference in modern contraception use between the current study and the others could be attributed to the differential health-related interventions, as different countries operate within diverse policy settings, rules, regulation and ideologies. The difference could also be due to the high educational attainment of the women surveyed and their husbands in the current study (Table 1) and to the reasons given by the women for using modern contraception, which were to help ensure proper care of the child and to prevent pregnancy or to ensure birth spacing (Figure 3). Among the modern contraceptives, injectables were used most (46.2%), while condoms (5.4%) and intrauterine devices (IUDs) (4.6%) were the least used. Injectable use in the current study is similar to what was reported in Zaria, Nigeria (41.5%) [18].

Reasons for the high usage of injectables could be due to the fact that they are more comfortable and less expensive. Even though condom is far less expensive than injectables and sometimes totally free, the element of discomfort that goes along with its use could be a disqualifying factor. Also, injectables are replicable every 2-3 months unlike condoms that need to be engaged in every episode of sexual intercourse. Contraceptive pills were equally being used by a large proportion of the women probably as an emergency control pill. Fascinatingly, sterilization was not used by any of the women. The reason could be that married women might have the mind-set of having children in the course of the marriage, hence did not consider sterilization as an option due to its long duration of potentiality [20]. There is the need to critically consider some of these modern contraceptive methods such as condoms that offer double protection, thus against both pregnancy and sexually transmissible infections (STIs), which was hardly used by respondents in this study. Occupation and decision-making status at home were factors that influenced modern contraception use in the current study. Private employees were 0.20 times less likely to use some form of modern contraception compared to the unemployed. Also, women without problems in decision-making were 4 times more likely to use modern contraception compared to women with problems in decision-making at home. This is similar to a study conducted among women in Ethiopia in which women with high decision-making powers were associated with modern contraception use [21]. This could be due to the fact that women without problems in decision-making have the self-efficacy for being responsible for their health, hence take decisions regarding modern contraception usage. It could also be that their spouses are supportive in the decision-making process regarding use of modern contraception.

## Conclusion

The findings of the current study revealed that the current use of modern contraception among married women was low. Privately employed women were less likely to use some form of modern contraception compared to housewives, while women without problems relating to decision-making at home were more likely to use modern contraception. Health promotion interventions should be put in place to increase the use of modern contraception among married women in the Ho West district of Ghana, focusing on the privately employed and those with problems in decision-making at home.

**Limitations of the study:** some married women in the current study were illiterate, making it very difficult to explain some of the modern contraception terms in the ewe dialect to them. However, the use of an interpreter minimized this problem. Also, being a cross-sectional study, cause-effect relationships could not be ascertained.

### What is known about this topic

- Despite technological advancements in modern contraceptive methods, still unintended pregnancy is a worldwide problem that affects women, their families and the society as a whole;
- Promotion of modern contraception is one of the main targets of the sustainable development goals;
- The acceptor rate for modern contraception in Ho West district, Ghana is low.

### What this study adds

- The current usage of modern contraception is low;
- Private employees are less likely to use modern contraception;
- Women having no problem with decision-making at home are more likely to use modern contraception.

## Competing interests

The authors declare no competing interests.

## References

[1] Frini HO, Nabag WOM (2013). The knowledge and determinant factors of contraceptive use among married Sudanese Women. App Sci Report.

[2] Obwoya JG, Wulifan JK, Kalolo A (2018). Factors influencing contraceptive use among women in the Juba city of South Sudan. International Journal of Population Research.

[3] United Nations (2015). Trends in contraceptive use worldwide 2015.

[4] Lim SS, Allen K, Bhutta ZA, Dandona L, Forouzanfar MH, Fullman N (2016). Measuring the health-related Sustainable Development Goals in 188 countries: a baseline analysis from the Global Burden of Disease Study 2015. Lancet.

[5] Starbird E, Norton M, Marcus R (2016). Investing in family planning: key to achieving the sustainable development goals. Glob Health Sci Pract.

[6] Handady SO, Naseralla K, Sakin HH, Alawad AAM (2015). Knowledge, attitude and practice of family planning among married women attending primary health center in Sudan. Int J Public Health Res.

[7] Gore S, Katkuri S (2016). A study to assess contraceptive use among married women in urban and rural areas: a comparative study. Int J Reprod Contracept Obstet Gynecol.

[8] Adongo PB, Tambong PT, Azongo TB, Phillips JT, Sheff MC, Stone AE (2014). A comparative qualitative study on misconceptions associated with contraceptive use in southern and northern Ghana. Frontiers in Public Health.

[9] Ghana Health Service (2016). Family Health Division. Annual Report.

[11] Ho West District (2010). Population & Housing Census, descriptive analytical report; Ho west district. Ghana statistical service.

[12] Saluja N, Sharma S, Choudhary S, Gaur D, Pandey S (2011). Contraceptive knowledge, attitude and practice among eligible couples of rural Haryana. Internet J Health.

[13] Ghana Statistical Service (2014). District analytical report. Ho West District.

[14] Cochran (1977). Sampling Techniques.

[15] UNICEF (2013). Statistics at a glance Ghana UNICEF.

[16] National Institute of Population Research and Training (2008). 2006 Bangladesh urban health survey (UHS) Dhaka.

[17] Tilahun T, Coene G, Luchters S, Kassahun W, Leye E, Temmerman M (2013). Family Planning Knowledge, Attitude and Practice among Married Couples in Jimma Zone, Ethiopia. PLoS ONE.

[18] Ghana Statistical Service (2014). Ghana demographic and health survey.

[19] Aliyu AA, Shehu AU, Sambo MN, Sabitu K (2010). Contraceptive knowledge, attitudes and practice among married women in Samaru community, Zaria, Nigeria. East Afr J Public Health.

[20] Wang W, Staveteig S, Winter R, Allen C (2017). Women's marital status, contraceptive use, and unmet need in sub-Saharan Africa, Latin America and the Caribbean.

[21] Fallis A (2013). Factors Influencing the Utilisation of Family Planning Service. J Chem Inf Model.

[22] Bogale B, Wondafrash M, Tilahun T, Girma E (2011). Married women's decision-making power on modern contraceptive use in urban and rural southern Ethiopia. BMC Public Health.

